# The effect of intentional summer flooding for mosquito control on the nitrogen dynamics of impounded *Avicennia germinans* mangrove forests

**DOI:** 10.1038/s41598-024-52248-4

**Published:** 2024-01-25

**Authors:** H. J. Laanbroek, M. C. Rains, J. T. A. Verhoeven, D. F. Whigham

**Affiliations:** 1 Ecology and Biodiversity Group, Institute of Environmental Biology, Utrecht University, Utrecht, The Netherlands; 2 Department of Microbial Ecology, NIOO-KNAW, Wageningen, The Netherlands; 3 School of Geosciences, University of South Florida, Tampa, FL USA; 4 Smithsonian Environmental Research Station, Edgewater, MD USA

**Keywords:** Biogeochemistry, Hydrology

## Abstract

Coastal wetlands such as mangrove forests are breeding grounds for nuisance-causing insects. Rotational Impoundment Management (RIM) for mosquito control involves annual summer inundation of impounded mangrove forests with estuarine water during the summer half year. However, in addition to controlling mosquitos, RIM may change biogeochemical pathways. This study set out to investigate how RIM quantitatively affects physicochemical soil characteristics and potential nitrifying and denitrifying activities (PNA and PDA), which are key in the global nitrogen cycle. Before and after the implementation of RIM, soil samples were collected annually in habitats differing in size and abundance of black mangroves (*Avicennia germinans*) in an impoundment with RIM and in an adjacent impoundment with a more open connection to the lagoon. Compared to the non-managed impoundment, soil moisture content, total nitrogen and PDA increased, while salinity decreased after the start of annual summer flooding, but only in the dwarf habitat. In the sparse and dense habitats, total nitrogen and PDA increased independently of summer flooding, whereas soil moisture content and salinity were not affected by RIM. Labile organic nitrogen increased only in the RIM impoundment, irrespective of the habitat type. PNA was generally not affected with time, except in the dwarf habitat in the absence of intentional summer flooding where it increased. Changes in the non-managed impoundment adjacent to the RIM impoundment demonstrate the importance of groundwater exchange in linked ecosystems. The consequences of interventions in the management of mangrove impoundments and adjacent forests for the nitrogen budget are discussed.

## Introduction

Mangrove forests, which are situated at the interface between terrestrial and marine ecosystems in tropical and subtropical regions, offer a large number of ecosystem goods and services such as sources of firewood and timber, sinks of sediments, nutrients and contaminants, important breeding sites and nursery grounds for fish and other marine and terrestrial animals, as well as protection against coastal erosion, hurricanes and tsunamis^1^. World-wide, mangrove ecosystems are threatened by human activities such as land conversion for aqua- and agriculture, coastal development, pollution and overexploitation of mangrove resources^1^. In addition to these threats, mangrove forests are increasingly impacted by climate change, especially via rising sea levels^2,3^. The list of ongoing and future threats to mangroves provides justification for studies of the functioning of mangrove forests in response to environmental changes^4^.

Mangrove forests along the Indian River Lagoon on the Atlantic coast of Florida have been impounded for mosquito control^5^, with Rotational Impoundment Management (RIM) being implemented in one of the mosquito control impoundments in March 2009 to improve mosquito control and mangrove growth^6^. In RIM, dedicated pumps are used to flood the impoundment with estuarine surface water only during the summer mosquito reproduction season, and tidal exchange is re-established between the impoundment and estuary through culverts during the rest of the year. RIM management of mosquitoes resulted in increased mangrove cover from 59 to 74% of the total impoundment within five years^7^. In comparison, mangrove cover in an adjacent impoundment that had more hydrological exchange with the lagoon did not change over the same five-year period, and an adjacent area that was never impounded also did not change in mangrove cover over the same five-year period.

According to numbers presented by Alongi^4^ in his review on nitrogen cycling and mass balance in the world’s mangrove forests, the microbial process of denitrification is on average 25% of the total nitrogen output by these forests. The input flux of nitrite and nitrate is only 31% of the nitrogen necessary for the flux of denitrification. Hence, a larger part of the nitrite and nitrate required for denitrification must have been produced in situ from ammonium by the process of aerobic nitrification. These numbers show the importance of denitrification and aerobic nitrification for the degree of nitrogen accumulation in mangrove ecosystems. Despite the positive effects of RIM for mosquito density and mangrove expansion, it remains unclear if annual summer inundation would affect the biogeochemical pathways of nitrification and denitrification. Due to enhanced levels of ammonium and nitrate in the surface water of the central Indian River Lagoon^8^, annual summer inundation could enrich the mangrove forests with mineral nitrogen that in turn would stimulate nitrification and denitrification. Within-impoundment hydrology during RIM would potentially vary spatially, with areas that are topographically higher and therefore less frequently flooded being more impacted than areas that are topographically lower and hence more frequently flooded. Therefore, when annual summer inundation affects biogeochemical pathways, the effects of intentional summer flooding as triggered by RIM will be most notable at the higher and initially drier parts of the impoundment because soil waterlogging will stimulate the anaerobic process of denitrification and suppress the aerobic process of nitrification at these sites more than at the lower and initially wetter sites.

The effects of RIM on potential nitrification and denitrification activities were previously reported^9^ for two impoundments in the Indian River Lagoon—one with RIM and an adjacent impoundment with a more open hydrologic exchange with the estuary. Verhoeven et al. studied the two impoundments during the first year that RIM was employed by sampling them before and after flooding had occurred in the RIM impoundment^9^. Here we report results of measurements made in the same impoundments 4 and 5 years after RIM began. The subsequent sampling provides the opportunity to determine if important ecological processes change over time following hydrologic modification. A comparison between the RIM and adjacent impoundments provided the opportunity to discriminate the effects of RIM from other external effects. Measurements were made in three mangrove habitats (dwarf, sparse or dense) with differences in the density and size of mangroves along an elevation gradient^9,10^.

## Methods

### Study sites

Impoundment #23 (27° 32′ 58″ N, 80° 19′ 35″ W) and impoundment #24 (27° 33′ 9″ N, 80° 19′ 34″ W) are part of a series of mosquito control impoundments that were installed along the central east coast of Florida in the early 1950s^6^. Both impoundments are adjacent to Big Starvation Cove, which is part of the Indian River Lagoon. Mosquito control impoundments are bordered on three sides with a dike constructed with material from a perimeter ditch on the inside of the dike, and a ditch on the side that is the border between the impoundment and the adjacent upland. By the 1970s, flap-gated culverts were installed in the dikes to prevent excessive water levels and to reconnect the mosquito control impoundments with the estuary for better plant growth^6^.

Impoundment #23 is ~ 10 ha in size with ~ 450 m of shoreline. It was maintained with just the flap-gated culverts until its dike was breached in 1974 and two 75 cm diameter open culverts were installed sometime thereafter^11^. Since then, free tidal exchange between the Indian River Lagoon and impoundment occurs through the breach and two 75 cm diameter culverts^10^. We refer to impoundment #23 as the non-managed impoundment.

Impoundment #24 is ~ 30 ha in size with ~ 2600 m of shoreline. It was maintained with just the flap-gated culverts until a total of five 75 cm diameter open culverts were installed between 1985 and 1987^12^. To further reduce oviposition by mosquitos in summers, Rotational Impoundment Management (RIM) was introduced in March 2009. RIM consists of pumping lagoon water into the impoundment between March and September each year with target water levels being between 10 and 30 cm above the soil surface^9^. Since then, free tidal exchange between the Indian River Lagoon and impoundment #24 occurs though the five 75 cm diameter open culverts between October and February and lagoon water is pumped into impoundment #24 and flows freely out to the Indian River Lagoon through the five 75 cm diameter open culverts between March and September. We refer to impoundment #24 as the RIM impoundment.

### Hydrology

We used a combination of modeled tidal data from the adjacent Indian River Lagoon and measured water levels in the impoundments to determine the frequency and duration of inundation for each sampling location. The Indian River Lagoon parallels the Atlantic coast, sheltered by a series of barrier islands. There are just five inlets that connect it to the Atlantic Ocean, the closest one ~ 10 km away. Tidal amplitudes are small, with the total maximum annual range at the non-managed and the RIM impoundments being ~ 70 cm. There is free tidal exchange between the Indian River Lagoon and the non-managed impoundment year-round and between the Indian River Lagoon and the RIM impoundment between October and February. Mean ± SD elevations are − 0.05 ± 0.11 m above mean sea level and − 0.09 ± 0.09 m above mean sea level in the non-managed and the RIM impoundment, respectively. These are well-below the mean and higher tide elevations, so the non-managed impoundment tidally floods daily year-round, and the RIM impoundment tidally floods daily between October and February. Therefore, tidal data alone can serve as the first foundation for estimating water levels at any location within the impoundment during these times.

Tidal elevations in the Indian River Lagoon adjacent to the impoundments were modeled on 1-h time intervals throughout the duration of the study using XTide 2 (http://www.flaterco.com/xtide/index.html), which uses station-specific harmonic constants based on local tide gauge data resulting in predictions accurate to ± 1 min and ± 0.03 m of measured high and low tides assuming no episodic storm surge. Station-specific harmonic constants were taken from Ankona, located 20 km to the south. Water levels were automatically measured on 15-min time intervals with a pressure transducer/datalogger at three locations in the non-managed impoundment and two locations in the RIM impoundment from October 2008 through September 2009 (Supplementary Figure S1). While pumping occurred in the RIM impoundment, water level elevations also were automatically measured on 1-min time intervals with a pressure transducer/datalogger adjacent to the pump station in the RIM impoundment (Supplementary Figure S1). These modeled and measured tidal and water elevations were used to estimate inundation duration at all sampling plots in both impoundments throughout the duration of the study.

Between October and February, mean daily water levels in both impoundments are highly correlated with mean daily water levels in the Indian River Lagoon, with *r* = 0.73 and 0.87 for the non-managed and the RIM impoundment, respectively. However, evapotranspiration lowers water levels in the impoundments more than in the adjacent Indian River Lagoon, so natural water levels are consistently ~ 35 cm lower in the impoundments than in the adjacent Indian River Lagoon^13^. Therefore, for the non-managed impoundment, a water level record for the duration of the study was created by projecting modeled the tidal elevation of − 35 cm throughout the impoundment. On daily time steps, these projected modeled water levels adequately represented the average measured water levels in the three piezometers (*NSE* = 0.26) in the non-managed impoundment (Supplementary Figure S2). Similarly, for the RIM impoundment, a water level record between October and February was created by projecting modeled tidal elevations − 35 cm throughout the impoundment. Again, on daily time steps, these projected modeled water levels adequately represented the average measured water levels in the two piezometers (*NSE* = 0.52). The modeled water levels in the impoundments were then projected on the sampling plots, for which the ground surface elevation had been surveyed. From this, the number of inundation hours or days per period for each sampling location were calculated. A period started in March when summer pumping also started, to March the following year.

### Vegetation

Vegetation in both impoundments is dominated by black mangrove (*Avicennia germinans*) with scattered white mangrove (*Laguncularia racemosa*) in the interior and red mangrove (*Rhizophora mangle*) at the fringe adjacent to the perimeter ditch at the inner side of the dike^10^. Areas dominated by *A. germinans* are characterized by a gradient of tree heights and densities that has served as a basis for the classification of the three habitats (dwarf, sparse, dense) that were sampled in this study. Characteristics of the three habitats are described by Feller et al.^10^. In addition to the black mangroves, all three areas had a few other salt-tolerant species, especially saltwort (*Batis maritima*). In the RIM impoundment, there were areas (salt pans) that had no vegetation or scattered dwarf mangroves and *B. maritima* prior to the start of RIM in March 2009.

### Collection of soil samples

Soil samples were collected in early March in 2008, 2009, 2013 and 2014 in both impoundments upon the start of summer pumping in the RIM impoundment, except in March 2008 when no pumping occurred. In each impoundment, 15 plots, each 1 m^2^, were established (5 in each of the three habitats). Sampling plots were chosen according to a stratified random selection within vegetation types^10^, but independent of soil elevation (Supplementary Figure S1). No noteworthy differences in soil texture were observed for the different sampling locations (Supplementary Table S1). At every sampling, 15 locations were visited in each impoundment*.* 5 in the dwarf, 5 in the sparse and 5 in the dense habitat zones. Because of the time required to process the soil samples, the two impoundments were sampled at two- to three-day intervals. At each sampling location, 3 soil cores were collected from the surface in aluminum tubes (3.9 cm diameter and 10 cm long), which were sealed with rubber stoppers at both ends and transported within 4 h to the Smithsonian Marine Station (SMS) at Fort Pierce located 16 km from the sampling locations.

To examine whether the biogeochemical and physicochemical values obtained from sampling in March, *i.e.*, at the start of summer pumping, reflect the values created by summer pumping 6 months prior to the sampling campaigns in March, we also collected and processed soil samples in November 2013 and compared these with values obtained from samples collected in March 2014. There were no significant differences in PNA and PDA between these sets of samples (Supplementary Figures S3, S4). This is in line with the absence of significant differences in soil moisture content between these months (Supplementary Figure S5).

### Processing of soil samples

Immediately upon arrival at the SMS, soil cores were prepared for further analyses and biogeochemical measurements. One of the three cores collected at each sampling location was used for the determination of salinity, pH, and dissolved nutrients. From this core, pore water was extracted by applying suction under vacuum condition as described by Verhoeven et al.^9^. The upper 5 cm of the remaining two cores was combined, mixed by hand, and divided into 6 subsamples for further analyses. Subsample 1 (20 g) was dried at 70 °C for 48 h for the determination of soil dry weight. The dried soil was stored for later analyses of total carbon and nitrogen. Samples of freeze-dried soils were also used for the determination of particle size distribution. Subsample 2 (20 g) was extracted with 50 ml of a 1 M KCl solution for one hour on a rotary shaker at 150 rpm for the determination of ammonium, nitrate, and phosphate contents; the extracts were refrigerated. Subsample 3 (20 g) was transferred to a 100 ml septum bottle and flushed with nitrogen for the determination of labile organic nitrogen. Of the nitrogen gas phase, 29 ml was replaced by 29 ml Instant Ocean Sea water of the same salinity as the corresponding pore water. After flushing the septum bottles with nitrogen, the soil slurries were incubated for 4 days at 40 °C in the dark, after which the slurry was decanted, mixed with 50 ml of a 1 M KCl solution, shaken for 1 h at 150 rpm and centrifuged for 30 min at 2500 rpm in the dark. The supernatants were used for the determination of ammonium as a measure of labile organic nitrogen. Subsamples 4 and 5 (20 g each) were used for the determination of potential nitrifying and denitrifying activities, respectively, as described previously^9^. Finally, a subsample of 5 g was freeze-dried at − 20 °C for future DNA-based microbial community analyses.

### Chemical analyses

Total soil carbon and nitrogen analyses were performed on a Gerhardt Kjeldatherm KB 40S destruction block. Nutrients present in extracted pore water samples, among which ammonium, nitrate, and phosphate, were measured on an Inductively Coupled Plasma Optical Emission Spectrometer 6300 (Thermo Fisher Scientific, Breda, the Netherlands). Concentrations of ammonium, nitrate and phosphate obtained from 1 M KCl soil extractions were determined on a Skalar Analytical Continuous Segmented Flow Analysis System SA2000/4000 (Skalar, Breda, the Netherlands). Amounts of nitrite and nitrate acquired from potential nitrifying activity measurements were measured on a QuAAtro Seal Autoanalyzer (Beun-De Ronde, Abcoude, the Netherlands). Concentrations of nitrous oxide that are required for the calculation of potential denitrifying activity, were measured by Electron Capture gas chromatography (Agilent Technologies, Amstelveen, the Netherlands). Freeze-dried soil samples were ground at 20 rpm using a ball mill (Retsch GmbH, Haan, Germany) and subsequently used for the determination of total nitrogen by means of an element CN analyzer (InterScience BV, Breda, the Netherlands). Particle size distribution in freeze-dried soil samples was determined on a Malvern particle size analyzer (Malvern Panalytical Ltd, Malvern, UK) at the Royal Netherlands Institute for Sea Research (NIOZ) in Yerseke, the Netherlands.

### Statistical analysis

Statistical analyses of modelled annual inundation days, measured abiotic soil characteristics and potential activities were performed with the Paleontological Statistics (PAST) software package^14^ version 4.03. PAST also was used to eliminate outliers, to confirm normality of data, to test for significant differences in values between impoundments, habitat zones, and years of sampling. The analyses were performed for the three mangrove habitat types separately.

## Permission to conduct research and to collect samples

The research was conducted in publicly owned impoundments where the Smithsonian Institution has conducted research for years. The St Lucie County Mosquito Control District, the organization that controls access to the impoundments provided keys to locked gates to the Smithsonian scientists who conducted the research.

## Results

### Number of annual inundation days

**Figure 1:**
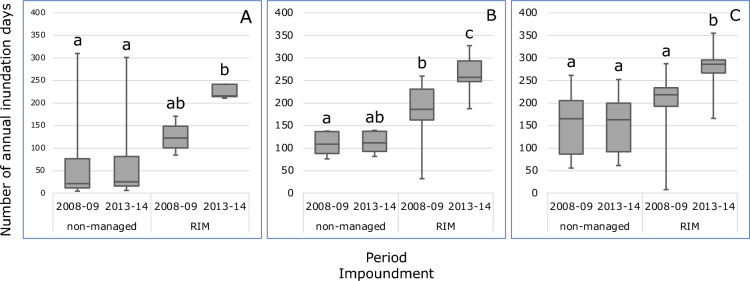
Boxplots of annual inundation days calculated for the periods 1 (2008–2009) and 2 (2013–2014) for the sampling locations in the dwarf (**A**), sparse (**B**) and dense (**C**) *Avicennia germinans* mangrove habitats present in the non-managed impoundment (#23) and the RIM impoundment (#24). Different characters indicate significant differences (*p* < 0.05, Dunn’s post hoc test) between median values.

Before the implementation of RIM, a significant difference in numbers of annual inundation days between both impoundments was only observed for the sparse habitat (Fig. 1). Pumping water from the Indian River Lagoon into the RIM impoundment increased the number of annual inundation days in each habitat of this impoundment, although only significantly in the dense habitat. In the period 2013–2014, the number of annual inundation days was significantly higher in all habitats of the RIM impoundment compared to the non-managed impoundment, where no changes in numbers of annual inundation days were observed in the three habitats. The large upper whisker observed in the period 2008–2009 and the period 2013–2014 in the dwarf habitat of the non-managed impoundment reflects the presence of a sampling site with a relatively low elevation of − 0.29 m (Supplementary Table S1). Nevertheless, this site belongs to the dwarf habitat type based on vegetation characteristics. The large lower whiskers in the period 2008–2009 in the sparse and dense habitat of the RIM impoundment reflect the presence of two sampling sites with relatively high elevations of 0.04 and 0.10 m, respectively (Supplementary Table S1).

### Physicochemical soil characteristics

Before the implementation of RIM, the largest significant differences in abiotic soil characteristics between both impoundments were observed in the dwarf habitat (Table 1). These differences encompassed significantly higher values for salinity, pore water nitrate, iron, magnesium, sodium, potassium, and sulfate were observed in the dwarf habitat of the RIM impoundment compared with the corresponding habitat in the non-managed impoundment. Several soil characteristics decreased significantly in time in all habitats of both impoundments, *i.e.,* extractable and pore water nitrate, calcium, and sodium (Table 1). Total N increased significantly in all habitats except in the dwarf habitat of the non-managed impoundment, whereas labile organic N increased significantly only in all habitats of the RIM impoundment. In the period 2013–2014, labile organic N was significantly larger in all habitats of the RIM impoundment compared to the corresponding habitats of the non-managed impoundment (Table 1). The same was observed for total N except for the dwarf habitat. Finally, salinity and dry bulk density became significantly lower, and moisture became significantly higher in the dwarf habitat of the RIM impoundment compared to the corresponding habitat of the non-managed impoundment.

**Table 1:** Spatial and temporal differences in median values of soil characteristics measured at individual sampling sites in different habitats in the *Avicennia germinans* impoundments (non-managed and RIM) in the periods 2008–2009 and 2013–2014. Different characters indicate significant differences between the values at the level of habitats.

Habitat	Impoundment	Period	Moisture	Dry bulk density	Salinity	pH	Total N	Labile organic N	Extractable NH_4_^+^	Extractable NO_3_^-^	Pore water NH_4_^+^	Pore water NO_3_^-^	Pore water Fe^2+^	Pore water Ca^2+^	Pore water Mg^2+^	Pore water Na^+^	Pore water K^+^	Pore water PO_4_^3-^	Pore water SO_4_^2-^
Dwarf	non-managed	2008- 2009	24.2 b	1.3 bc	50 a	7.7 a	0.7 ab	23.2 a	2.4 ab	1.7 b	0.4 b	0.9 b	0.1 a	0.8 b	1.5 a	18.5 a	0.5 a	0.3 ab	4.4 a
		2013- 2014	25.6 b	1.2 ab	43 a	7.8 a	1.1 bc	17.5 a	3.2 b	0.9 a	0.3 a	0.2 a	0.0 ab	0.1 a	1.4 a	11.2 a	0.4 a	0.2 a	4.3 ab
	RIM	2008–2009	22.8 a	1.4 c	74 b	7.6 a	0.5 a	22.5 a	1.1 a	2.2 b	0.3 ab	2.9 c	0.2 b	0.9 b	2.2 b	42.0 b	0.8 b	0.4 b	5.0 b
		2013–2014	33.2 c	1.2 a	52 a	7.7 a	1.8 c	90.3 b	11.0 ab	0.1 a	0.3 ab	0.2 ab	0.1 a	0.5 a	1.5 a	12.9 a	0.4 a	0.6 b	4.1 ab
Sparse	non-managed	2008–2009	25.0 a	1.3 b	63 a	7.5 a	0.6 a	27.2 a	2.5 a	1.8 b	0.3 b	1.0 b	0.12 a	0.8 b	1.5 ab	16.0 b	0.5 c	0.3 b	4.3 a
		2013–2014	30.8 ab	0.9 a	44 a	7.6 a	1.2 b	28.5 a	2.0 a	0.1 a	0.2 ab	0.2 a	0.1 a	0.5 a	1.3 a	10.6 a	0.4 a	0.2 ab	4.3 a
	RIM	2008–2009	36.0 ab	1.0 a	55 a	7.5 a	1.3 b	42.5 a	1.3 a	5.0 b	0.2 a	1.1 b	0.2 a	0.9 b	1.8 b	23.7 b	0.5 bc	0.2 b	4.3 a
		2013–2014	44.3 b	0.9 a	50 a	7.6 a	2.7 c	86.0 b	7.8 a	0.1 a	0.3 b	0.1 a	0.1 a	0.5 a	1.5 ab	12.2 a	0.4 ab	0.1 a	5.2 a
Dense	non-managed	2008–2009	39.0 a	1.0 a	58 a	7.4 a	2.0 a	52.6 a	3.5 a	1.9 b	0.4 b	0.8 b	0.2 a	1.0 b	2.0 ab	30.3 b	0.6 c	0.5 b	5.7 a
		2013–2014	43.0 ab	0.7 a	39 a	7.5 a	2.6 b	55.6 a	3.9 a	0.0 a	0.3 ab	0.2 a	0.2 a	0.5 a	1.4 a	10.9 a	0.4 a	0.3 ab	4.4 a
	RIM	2008–2009	47.0 ab	0.8 a	54 a	7.3 a	2.4 b	55.5 a	1.6 a	2.5 b	0.1 a	0.1 b	0.3 a	0.8 b	2.0 b	21.6 b	0.6 bc	0.0 b	5.7 a
		2013–2014	54.2 b	0.7 a	39 a	7.4 a	3.5 c	72.0 b	3.1 a	0.1 a	0.3 b	0.1 a	0.2 a	0.6 a	1.5 ab	11.2 a	0.4 ab	0.0 a	4.8 a

Units used: Moisture = percent; dry bulk density = g/cm^3^; salinity = per mille; pH = none; total nitrogen = mg/g dry soil. labile organic nitrogen = mg N/kg dry soil; extractable ammonium and nitrate = mg N/kg dry soil; pore water ammonium. nitrate. iron and phosphate = mg/liter; pore water calcium. magnesium. sodium. potassium and sulfate = g/liter.

The first two principal components derived from a Principal Component Analysis performed on the soil characteristics, explained 30.6% and 20.1%, respectively, of the variance between the samples (Supplementary Figure S6). Principal Component 1(PC1) was largely determined by salinity and related soil components, *i.e.*, pore water potassium, sodium, and calcium (all positively), while Principal Component 2 (PC2) was mostly affected by moisture content, total N, and labile organic N (all positively) and by dry bulk density and pH (both negatively). The number of annual inundation days was significantly and negatively correlated with PC1, and positively with PC2 (Supplementary Table S2).

### Potential nitrifying activities

**Figure 2:**
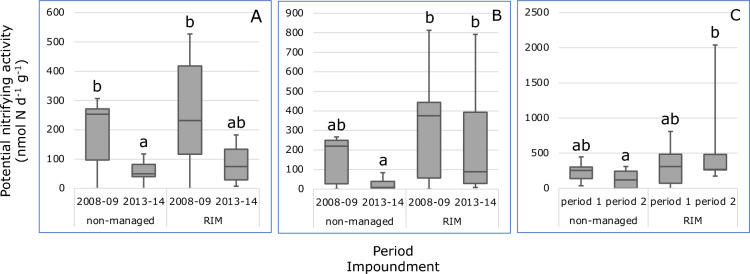
Boxplots of potential nitrifying activities measured in soil samples measured in samples collected in the dwarf (**A**), sparse (**B**) and dense (**C**) *Avicennia germinans* mangrove habitats in the non-managed impoundment (#23) and in the RIM impoundment (#24) in the periods 2008–2009 (period 1) and 2013–2014 (period 2). Different characters indicate significant differences (*p* < 0.05, Dunn’s post hoc test) between median values. *Note* 1 outlier was omitted from period 1in the dwarf habitat of the non-managed impoundment, 1 outlier from period 2 in the dwarf habitat of the RIM impoundment, 2 outliers from period 2 in the sparse habitat of the RIM impoundment, and 1 outlier from period 2 in the dense habitat of the RIM impoundment.

No significant differences in potential nitrifying activities (PNA) were observed for the mangrove habitats before the implementation of RIM (Fig. 2). PNA decreased temporally in most habitats in both impoundments, but the decrease was only significant in the dwarf habitat of the non-managed impoundment. In the period 2013–2014, PNA values were significantly higher in the sparse and dense habitats of the RIM impoundment compared to the corresponding habitats of the non-managed impoundment. No significant differences in PNA between both habitats were observed for the dwarf habitat after the onset of RIM.

PNA was significantly and positively correlated with the number of annual inundation days (Supplementary Table S2).

### Potential denitrifying activities

**Figure 3:**
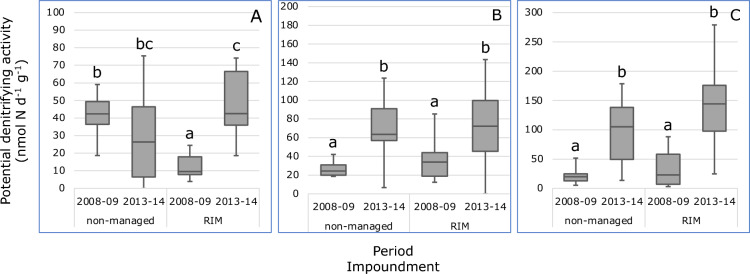
Boxplots of potential denitrifying activities measured in soil samples measured in samples collected in the dwarf (**A**), sparse (**B**) and dense (**C**) *Avicennia germinans* mangrove habitats in the non-managed impoundment (#23) and in the RIM impoundment (#24) in the periods 2008–2009 (period 1) and 2013–2014 (period 2). Different characters indicate significant differences (*p* < 0.05, Dunn’s post hoc test) between median values. *Note* 1 outlier was omitted from period 2 in the dwarf habitat of the RIM impoundment, and 1 outlier from period 1 in the dense habitat of the non-managed impoundment.

Before the implementation of RIM, the potential denitrifying activities (PDA) in the dwarf habitat of the RIM impoundment were significantly lower than in the corresponding habitat in the non-managed impoundment, whereas for the other habitat types no significant differences in PDA were observed between both impoundments (Fig. 3). After the onset of RIM, PDA increased in all habitats in both impoundments and the increases were significant, except in the dwarf habitat in the non-managed impoundment. In 2013–2014, no significant differences in PDA between impoundments were measured in any of the three habitats.

PDA was significantly and negatively correlated with PC1 of the Principal Component Analysis (Supplementary Table S2). No significant correlation was observed between PDA and PNA, and between PDA and the number of annual inundation days.

## Discussion

**Figure 4:**
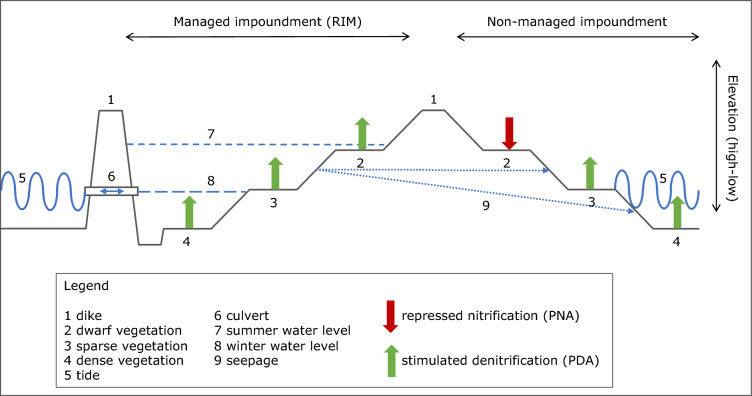
A graphical abstract summarizing significant changes in potential nitrifying and denitrifying activities observed in the different *Avicennia germinans* mangrove habitats in the RIM impoundment and in the non-managed impoundment after the onset of RIM. Summer water level was established by pumping estuarine surface water into the impoundment.

In general, potential denitrifying activities (PDA) increased with time irrespective of the implementation of RIM, while potential nitrifying activities (PNA) remained more constant in time and were unaffected by the change in hydrology. As shown graphically in Fig. 4, PDA increased in all habitats, except for the dwarf habitat in the non-managed impoundment. In contrast, PNA decreased only significantly in the same habitat after the onset of RIM. The increases in PNA may have been due to an increase in soil moisture content that was measured in all habitats after the start of RIM (Supplementary Figure S7). Increases in PNA suggest that both carbon and nitrate were not limiting denitrification in their capacity of electron donor and acceptor, respectively. Hence, the presence of aerobic conditions likely restricted denitrification before the onset of RIM. The significant negative correlation of PDA with Principal Component 1, which was largely determined by salinity related variables, suggests that the observed decrease in salinity after the implementation of RIM also may have been a cause of increased PDA values. Measurements of the effect of salinity on PDA in estuarine soils have been rare. Wang and colleagues measured a significant and negative correlation between PDA and salinity across a salinity gradient of 0–30 PSU in soil slurries^15^, which is in line with our results, although some habitats in the RIM impoundment were more saline than 30 PSU before RIM started. Hence, PDA might have been relieved from salinity stress in both impoundments due to the implementation of RIM.

A possible explanation for the similarity between the impoundments with respect to changes in PDA, is seepage of water during the periods of inundation from the RIM impoundment to the adjacent non-managed impoundment under the earthen dike that is shared by both (Fig. 4). The earthen dike is narrow and was constructed to be wide enough to allow vehicle access. The dike is also a surface feature that was placed on top of otherwise undisturbed native sediments comprised of high-permeability sands^13^. When inundated, water levels in the RIM impoundment are variable but typically tens of centimeters higher than in the adjacent impoundment. This creates a steep hydraulic gradient, driving groundwater under the narrow earthen dike through the shared native sediments. The effects of this seepage from the RIM to the adjacent non-managed impoundment are evident in groundwater levels in the two impoundments (Supplementary Figure S8). At the start of pumping lagoon water in March 2009, groundwater levels in the RIM impoundment increased immediately from below to above mean sea level. At the same time, groundwater levels in the adjacent non-managed impoundment also increased, but at a slower rate. Moreover, groundwater levels in this latter impoundment started to initially increase at the sites closest to the shared earthen dike and only later at sites more distant from the dike. This type of groundwater connectivity, including the lag in the response related to distance, is consistent with previous studies of groundwater connectivity between this same RIM impoundment and the adjacent Indian River Lagoon^13^. Moreover, soil moisture content increased in both impoundments after pumping water into the RIM impoundment began (Supplementary Figure S7). Increased soil moisture in both impoundments also resulted in a decrease in bulk density in most habitats (Supplementary Figure S9). This an example of the importance of connectivity between different habitats that are hydrologically connected. Hydrologic conditions in both impoundments were affected by managed inundation in the RIM impoundment alone. Rapid and geographically extensive groundwater connectivity is a hallmark of many wetland ecosystems, in Florida^16,17^ and throughout the world^18^. It also has been previously demonstrated at this site and across this system of earthen dikes^13^. Hydrological systems behave dynamically, in part in response to boundary conditions. Therefore, the effects of hydrologic management in one area may routinely be translated to adjacent areas. In our study, the higher parts of the non-managed impoundment, which are characterized by dwarf growth of the mangrove trees, are likely least affected by a groundwater flow from the RIM impoundment, and showed a behavior of PDA and PNA that was opposite to all other impoundments.

Denitrification equates to 35% of total N input in world’s mangrove ecosystems, so contributing to 0.5–2.0% to the global coastal ocean (Alongi 2020). Hence, an increase in denitrification by application of RIM will increase the loss of nitrogen from impounded mangrove forest with possible implications for their productivity, but also for the productivity of adjacent non-managed mangrove forests that are connected by subsurface water flows. This is certainly a consideration when carrying out interventions in the management of impounded mangrove forests. In contrast to PDA, PNA seems to be less affected by the implication of RIM. Only the dwarf habitat at higher elevations in the non-managed impoundment showed a significant decrease in time. Since this habitat seems not to be affected by intentional summer flooding in the adjacent RIM impoundment, an unknown factor must have been the cause of such a decrease.

Among the changes that occurred simultaneously in both impoundments after the implementation of RIM was the increase of total nitrogen in all habitat types (Supplementary Figure S10). In his review on nitrogen cycling in mangrove forests, Alongi mentions a number of mechanisms leading to conservation of nitrogen in mangrove forest soils^4^, *i.e.* (1) a highly efficient solute uptake by trees and microbes, (2) a high nitrogen-use efficiency and high rates of leaf resorption, (3) low rates of nitrogen loss such as dissolved nitrogen export and nitrous oxide emissions in proportion to nitrogen inputs, (4) export of highly refractory nitrogen in the form of humic and fulvic acids, (5) rapid rates of nitrogen fixation at the soil surface and on various forest components such as bark and pneumatophores, and (6) large belowground reservoirs of dead roots. All these mechanisms may have played a role in the accumulation of nitrogen in both impoundments. Interestingly, a large difference between both impoundments is observed in the behavior of labile organic nitrogen. Whereas labile organic nitrogen increased significantly in the habitats of the RIM impoundment after the implementation of summer pumping in March 2009, no significant changes were observed in the habitats of the non-managed impoundment (Supplementary Figure S11). The increases in labile organic nitrogen were most pronounced in the dwarf and sparse habitats of the RIM impoundment, which is in agreement with the observed stimulation of plant growth in these habitats of the impoundment after the onset of RIM^7^. Hence, the increase in labile organic nitrogen seems to be coupled to plant growth. Oostdijk et al.^7^ suggested that a relief of salt stress likely stimulated mangrove growth in the RIM impoundment. After the implementation of RIM, salinity decreased in all habitats of both impoundments, (Supplementary Figure S12).

Although interesting, a direct comparison of our present data on the impact of RIM on nitrogen-related soil characteristics with those of our former study^9^ is difficult to make due to the presence of only one year after the implication of RIM in the latter study. Nevertheless, looking at differences in trends observed in the RIM impoundment is possible. Increases and decreases in soil characteristics that were detected in 2010 being one year after the start of annual summer flooding, either continued, stabilized, or disappeared again in later years. In this way, the increase in PDA observed in all habitats stabilized in the dwarf habitat but continued in the sparse and dense habitats, the rise in soil moisture content stabilized in all habitats, and the increase in pore water ammonium disappeared again after 2010 in all habitats. The decline in salinity noticed in 2010 in all habitats continued to decrease in the dwarf habitat but stabilized in the other habitats. The decreases in PNA and pore water nitrate noticed in 2010 continued to decrease in the years thereafter in all habitats. On the other hand, changes in soil characteristics that had not been detected in 2010, started to change in later years. So, after 2010, labile organic nitrogen started to increase in all habitats, while total soil nitrogen and extractable ammonium started to decrease in all habitats. Hence this comparison of data between years makes it clear that conclusions about changes after the intervention in the hydrological regime should not be drawn too early.

Since the processes of nitrification and denitrification affect the nitrogen output of mangrove ecosystems, the results of this study have additional global implications should similar management approaches be applied in other areas where non-chemical approaches might be used to manage nuisance-causing insects. However, it is important to stress that changes in management meant for a restricted area, such as the application of RIM, may have a larger spatial impact through the movement of subsurface water.

### Supplementary Information

Supplementary Information.

## Data Availability

The datasets generated during and/or analyzed during the current study are available in the Marine Data Archive repository, https://mda.vliz.be/, or can be requested by contacting the corresponding author.
