# Supplementary material for: The effect of intentional summer flooding for mosquito control on the nitrogen dynamics of impounded Avicennia germinans mangrove forests
Source: Sci Rep. 2024 Jan 25;14:2165. doi: 10.1038/s41598-024-52248-4 (PMC10811325; doi:10.1038/s41598-024-52248-4)
Supplement: Supplementary file 1 — Supplementary Information. [file 41598_2024_52248_MOESM1_ESM.pdf]

The effect of intentional summer flooding for mosquito control on the nitrogen dynamics of an impounded *Avicennia germinans* mangrove forest

H.J. Laanbroek[1.2.4*]. M.C. Rains[3]. J.T.A. Verhoeven[1.4]. D.F. Whigham[4]

[1] Ecology & Biodiversity Group. Institute of Environmental Biology. Utrecht University. the Netherlands

[2] Department of Microbial Ecology. Netherlands Institute of Ecology (NIOO-KNAW). Wageningen. the Netherlands

[3] Ecohydrology Research Group. University of South Florida. Tampa. FL. USA

[4] Smithsonian Environmental Research Station. Edgewater. MD. USA

*Corresponding author: h.j.laanbroek@uu.nl

Other e-mail addresses:

MR: mrains@usf.edu

JTAV: j.t.a.verhoeven@uu.nl

DFW: whighamd@si.edu

Supplementary Table S1: Physical soil properties of the *Avicennia germinans* mangrove sampling sites at North Hutchinson Island. Florida. USA.

Sampling site	Impoundment	Habitat	Geography			Particle size distribution						
			Latitude	Longitude	Elevation (m)	Median grain size (µm)	Specific surface area (cm/ml)	% silt (<63 mµ)	% very fine sand (63 - 125 µm)	% fine sand (125 - 250 µm)	% medium sand (250 - 500 µm)	% coarse sand (500 - 1000 µm)
23.01	Non-managed	Dwarf	27° 32' 49.37"	-80° 19' 39.05"	-0.29	171	0.20	11	22	35	20	13
23.02	Non-managed	Dwarf	27° 32' 53.67"	-80° 19' 35.06"	-0.01	229	0.12	4	16	34	28	17
23.03	Non-managed	Dwarf	27° 33' 03.09"	-80° 19' 30.71"	0.06	188	0.14	5	19	44	21	11
23.04	Non-managed	Dwarf	27° 33' 03.33"	-80° 19' 31.59"	0.08	253	0.11	4	11	34	30	21
23.05	Non-managed	Dwarf	27° 33' 03.32"	-80° 19' 31.91"	0.11	245	0.11	4	12	35	28	21
23.06	Non-managed	Sparse	27° 32' 55.59"	-80° 19' 36.34"	-0.01	173	0.19	8	22	40	20	10
23.07	Non-managed	Sparse	27° 32' 55.87"	-80° 19' 34.78"	-0.02	366	0.10	5	7	19	36	32
23.08	Non-managed	Sparse	27° 33' 03.12"	-80° 19' 33.36"	-0.04	224	0.13	5	16	34	25	20
23.09	Non-managed	Sparse	27° 32' 52.71"	-80° 19' 36.24"	-0.07	205	0.12	4	17	40	27	12
23.10	Non-managed	Sparse	27° 32' 49.63"	-80° 19' 38.47"	-0.07	289	0.09	3	10	30	31	26
23.11	Non-managed	Dense	27° 32' 55.27"	-80° 19' 37.65"	-0.02	171	0.18	8	23	39	17	13
23.12	Non-managed	Dense	27° 33' 02.24"	-80° 19' 35.58"	-0.10	235	0.19	11	14	28	28	20
23.13	Non-managed	Dense	27° 32' 56.33"	-80° 19' 36.73"	0.01	151	0.25	13	26	40	15	7
23.14	Non-managed	Dense	27° 32' 50.38"	-80° 19' 39.24"	-0.15	201	0.16	8	19	34	23	17
23.15	Non-managed	Dense	27° 32' 49.37"	-80° 19' 38.93"	-0.22	202	0.20	11	17	32	25	15

Sampling site	Impoundment	Habitat	Geography			Particle size distribution						
			Latitude	Longitude	Elevation (m)	Median grain size (µm)	Specific surface area (cm/ml)	% silt (<63 mµ)	% very fine sand (63 - 125 µm)	% fine sand (125 - 250 µm)	% medium sand (250 - 500 µm)	% coarse sand (500 - 1000 µm)
24.01	RIM	Dwarf	27° 33' 05.39"	-80° 19' 34.41"	-0.03	189	0.14	4	18	46	24	8
24.02	RIM	Dwarf	27° 33' 05.86"	-80° 19' 31.72"	-0.03	236	0.14	6	11	36	31	15
24.03	RIM	Dwarf	27° 33' 06.15"	-80° 19' 32.73"	-0.02	254	0.14	7	9	32	34	16
24.04	RIM	Dwarf	27° 33' 06.78"	-80° 19' 32.64"	-0.08	185	0.16	6	18	47	24	5
24.05	RIM	Dwarf	27° 33' 14.41"	-80° 19' 29.48"	-0.08	177	0.17	6	22	42	19	11
24.06	RIM	Sparse	27° 33' 05.71"	-80° 19' 29.29"	0.04	209	0.13	5	15	41	27	12
24.07	RIM	Sparse	27° 33' 06.97"	-80° 19' 32.97"	-0.09	187	0.15	7	20	38	21	14
24.08	RIM	Sparse	27° 33' 23.31"	-80° 19' 35.31"	-0.21	160	0.17	7	26	45	15	7
24.09	RIM	Sparse	27° 33' 22.51"	-80° 19' 33.88"	-0.17	180	0.16	8	21	38	20	13
24.10	RIM	Sparse	27° 33' 14.07"	-80° 19' 30.89"	-0.11	169	0.17	9	23	40	19	9
24.11	RIM	Dense	27° 33' 08.00"	-80° 19' 32.84"	-0.13	180	0.14	7	22	40	21	11
24.12	RIM	Dense	27° 33' 22.87"	-80° 19' 37.53"	-0.16	153	0.25	15	24	36	16	9
24.13	RIM	Dense	27° 33' 22.34"	-80° 19' 37.01"	-0.25	195	0.21	12	17	33	24	14
24.14	RIM	Dense	27° 33' 06.98"	-80° 19' 36.16"	0.10	486	0.06	4	3	7	39	48
24.15	RIM	Dense	27° 33' 09.94"	-80° 19' 31.07"	-0.17	219	0.13	6	15	36	25	18

Supplementary Table S2: Spearman rank order correlations between annual inundation days and potential nitrification and denitrification potential (PNA and DNA. respectively) and the first two Principal Components 1 and 2 (PC1 and PC2) of a Principal Component Analysis based on soil characteristics measured in March 2008, 2009, 2013 and 2014. *p* values are presented above the diagonal and Spearman's rho values below the diagonal. Significant correlations are shown by asterisks.

	Number annual inundation days	PNA	PDA	PC 1	PC 2
Number annual inundation days		0.030*	0.533	0.012*	0.000*
PNA	0.198*		0.093	0.107	0.045
PDA	0.057	0.154		0.000*	0.114
PC 1	-0.229*	-0.148	-0.594*		0.875
PC 2	0.623*	0.183	0.145	0.014	

Note: PC1 and PC2 are explained in Supplementary Figure S1.

Supplementary Figure S1: The locations of the individual sampling sites in the dwarf (circles), sparse (triangles), and dense (squares) habitats of *Avicennia germinans* in the non-managed impoundment (green symbols) and in the RIM impoundment (orange symbols) at North Hutchinson Island in St Lucie County, Florida. In addition, the locations of the piezometers for measuring the frequency and duration of inundation have been indicated by blue starts (A23 – C23, A24 – B24). The location of the pumping station is shown by a red diamond. The locations have been drawn on a Hutchinson Island map created by Mapcarta (Mapcarta.com).

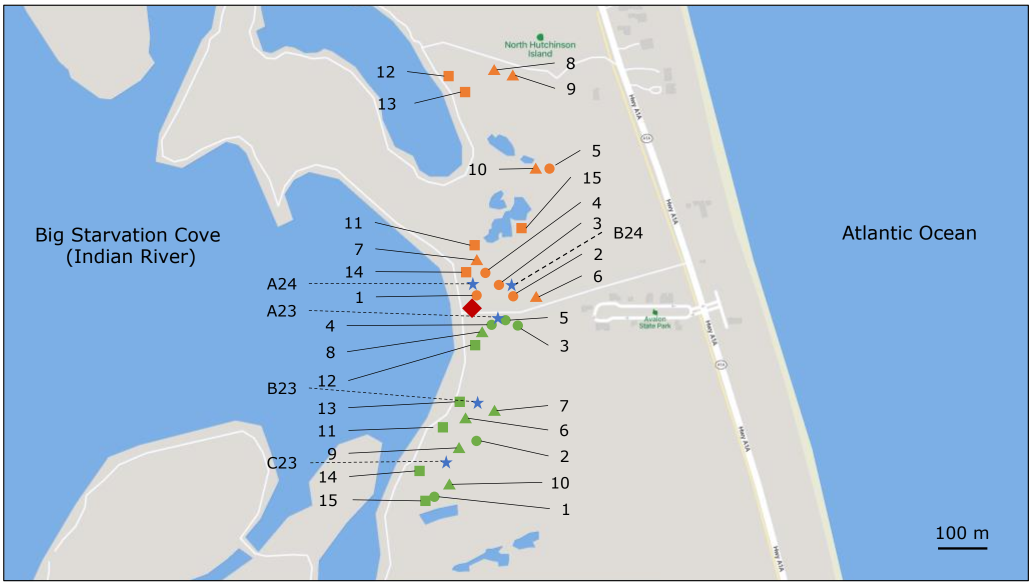

Supplementary Figure S2: Mean measured *versus* modeled groundwater levels on daily time steps in the non-managed (A) and in the RIM impoundment (B). For the non-managed impoundment, the *NSE* of 0.26 primarily reflected the overestimation of modeled water levels in the dry season (*i.e.*, January-May) when evapotranspiration is most important. For the RIM impoundment, the *NSE* is 0.52. The latter comparison does not extend into March-September, because the impoundment would be flooded during that time when operated as a RIM impoundment and therefore water levels would be measured not modeled during that interval.

A

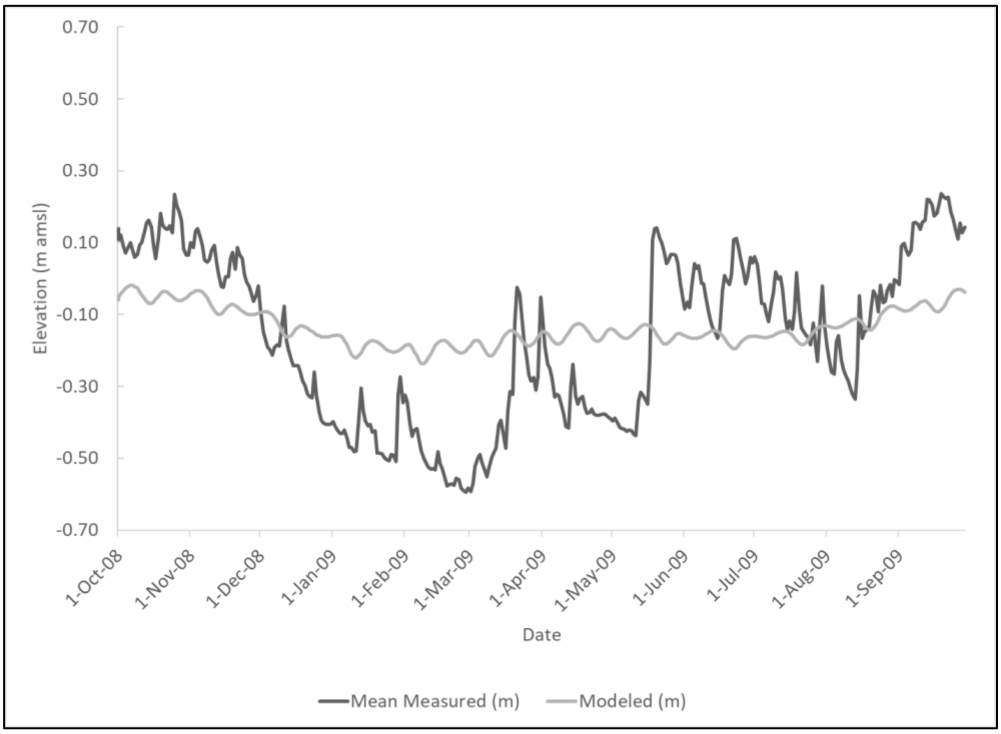

B

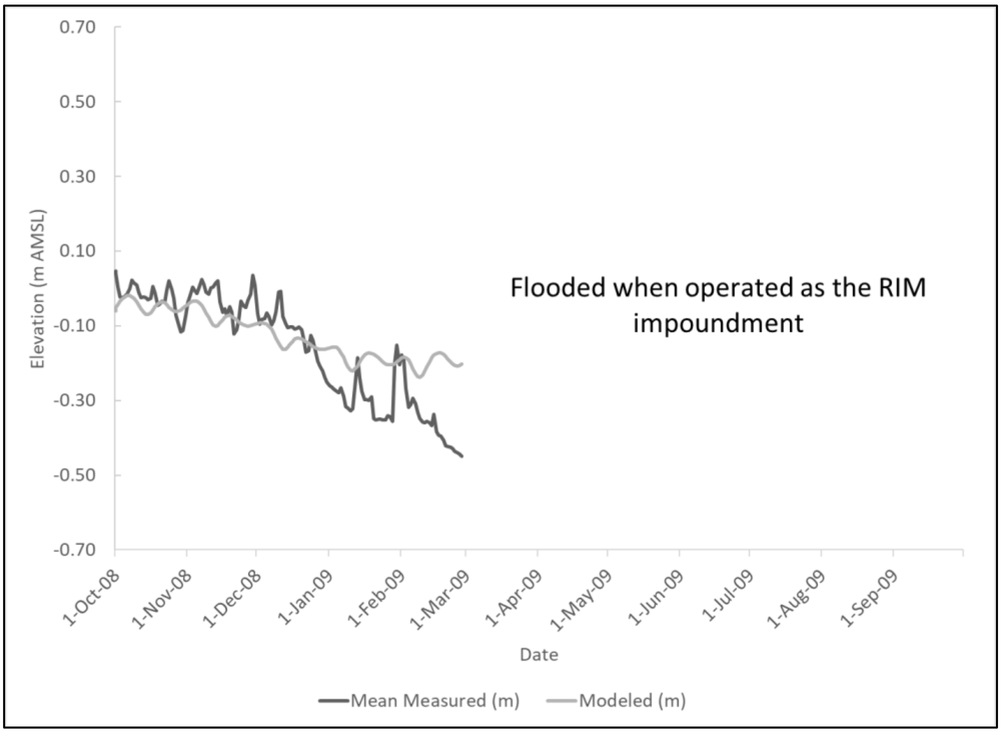

Supplementary Figure S3: Boxplots presenting Potential Nitrifying Activities (PNA) measured at the sampling sites in the dwarf (A), sparse (B) and dense (C) habitats in the non-managed and RIM impoundment in November 2013 (13-11) and in March 2014 (14-03). Different characters above the plots present significant differences ($p < 0.05$, Dunn's post hoc test) between the median values.

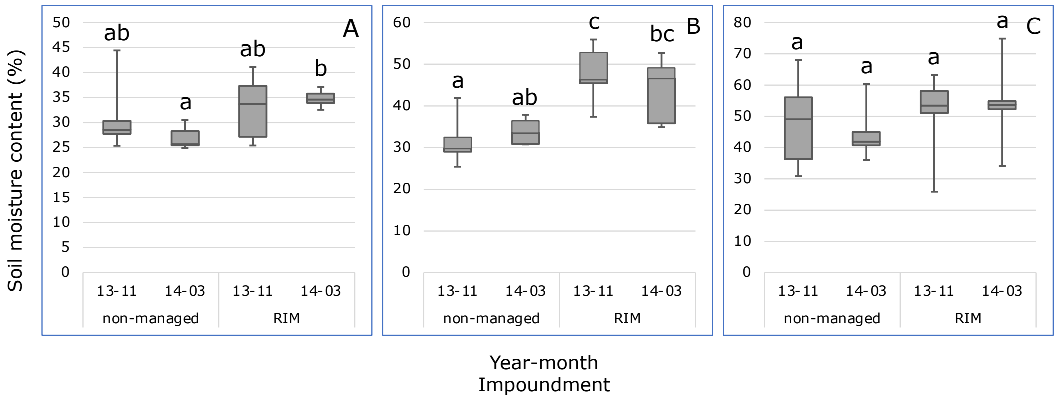

Note: No outliers were detected.

Supplementary Figure S4: Boxplots presenting Potential Denitrifying Activities (PDA) measured at the sampling sites in the dwarf (A), sparse (B) and dense (C) habitats in the non-managed and RIM impoundment in November 2013 (13-11) and in March 2014 (14-03). Different characters above the plots present significant differences ($p < 0.05$, Dunn's post hoc test) between the median values.

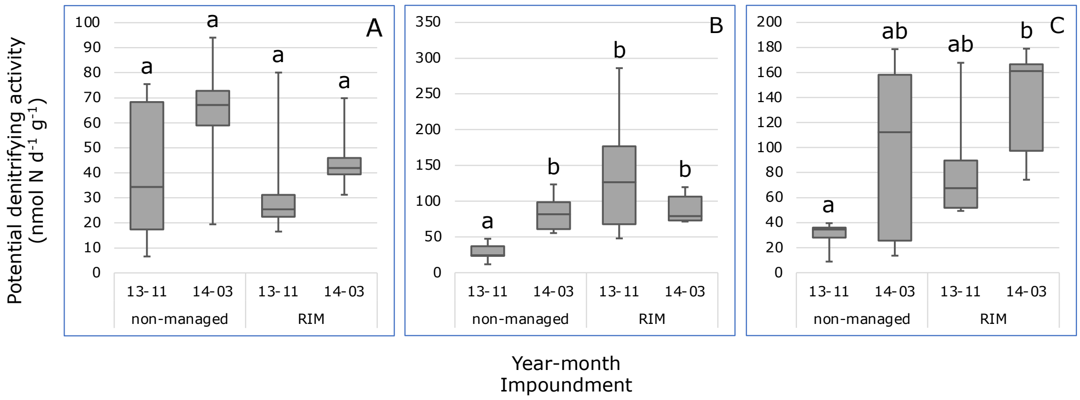

Note: No outliers were detected.

Supplementary Figure S5: Boxplots presenting soil moisture contents measured at the sampling sites in the dwarf (A), sparse (B) and dense (C) habitats in the non-managed and RIM impoundment in November 2013 (13-11) and in March 2014 (14-03). Different characters above the plots present significant differences ($p < 0.05$, Dunn's post hoc test) between the median values.

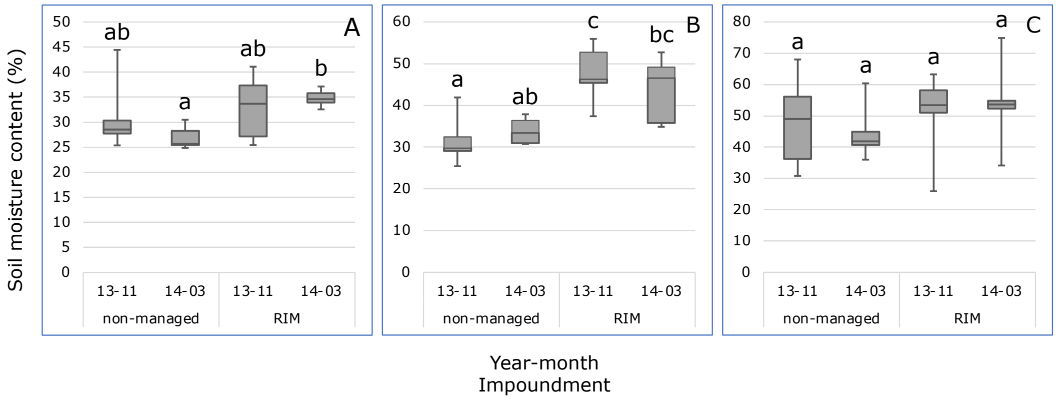

Note: No outliers were detected.

Supplementary Figure S6: Loading plot of vectors obtained from a Principal Component Analysis based on soil characteristics measured in samples collected in March 2008, 2009, 2013, and 2014. Soil characteristics presented in blue originate from pore water measurements.

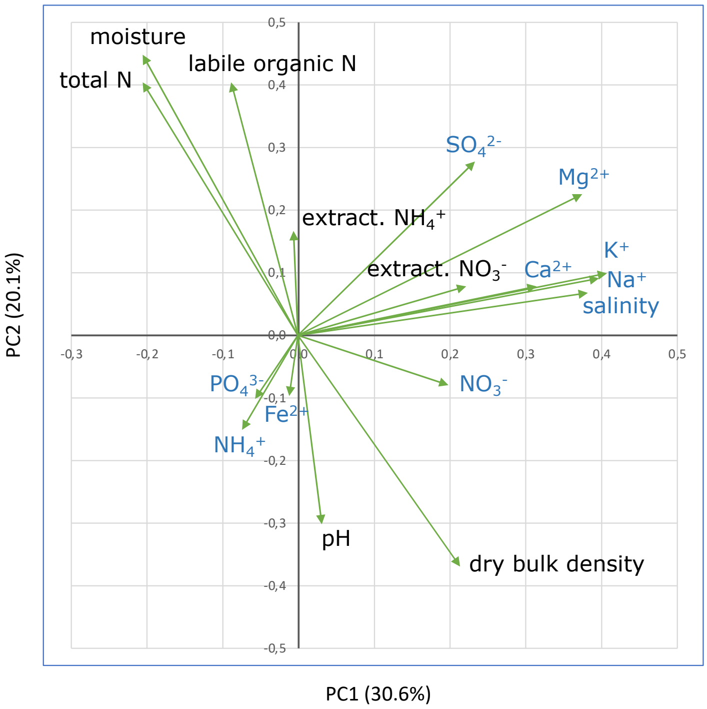

Supplementary Figure S7: Boxplots presenting soil moisture contents measured at the sampling sites in the dwarf (A), sparse (B) and dense (C) habitats in the non-managed and RIM impoundment in the periods 2008-2009 and 2013-2014. Different characters above the plots present significant differences (*p* < 0.05, Dunn's post hoc test) between the median values.

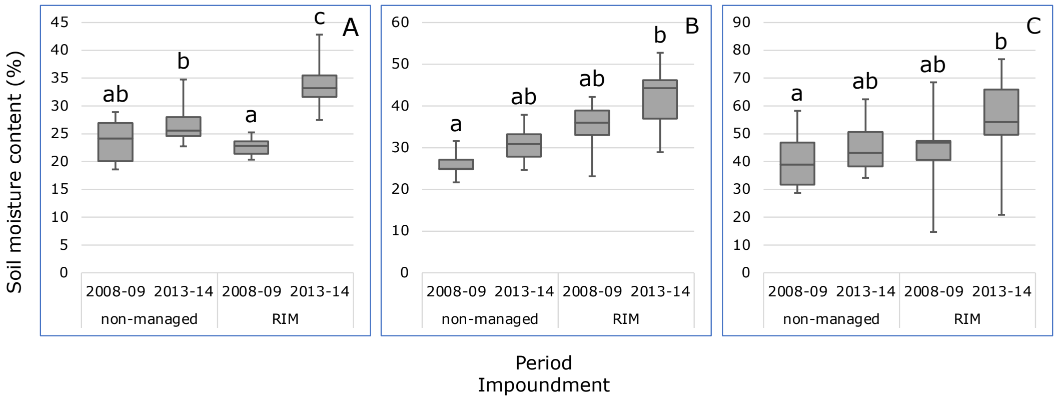

Note: No outliers were detected.

Supplementary Figure S8: Groundwater levels during the dry season in the non-managed impoundment (A, #23) and in the RIM impoundment (B, #24). Points 23-A, 23-B, and 23-C are positions on a transect that runs parallel to the Indian River Lagoon (IRL), from almost directly adjacent to the RIM-impoundment (23-A) to closer to the breach in the non-managed impoundment (23B then 23C). Points 24A and 24B are positions on a transect that runs perpendicular to the IRL, from near the IRL (24A) to the interior of the mangrove forest (24B).

A

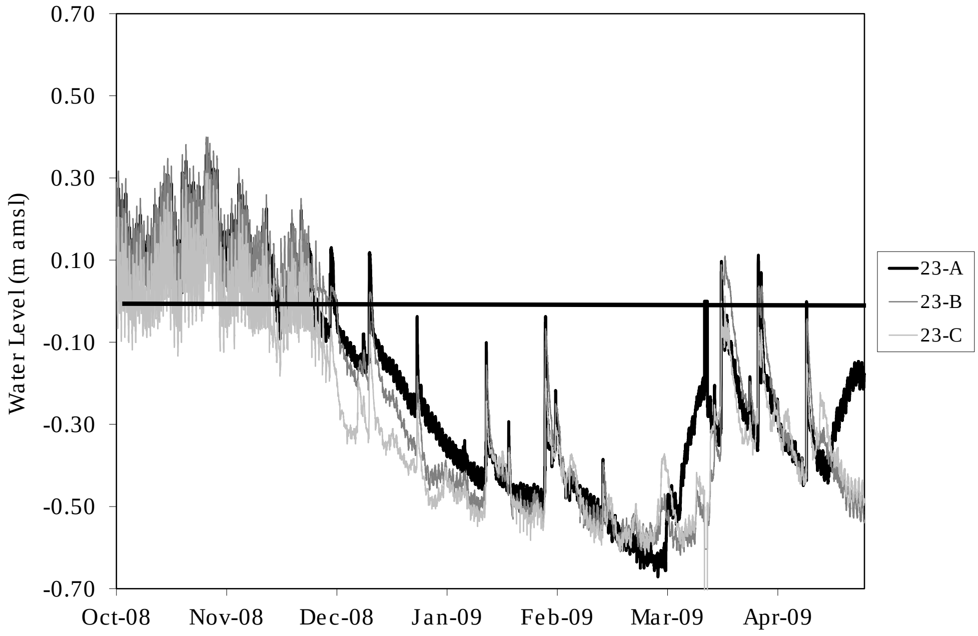

B

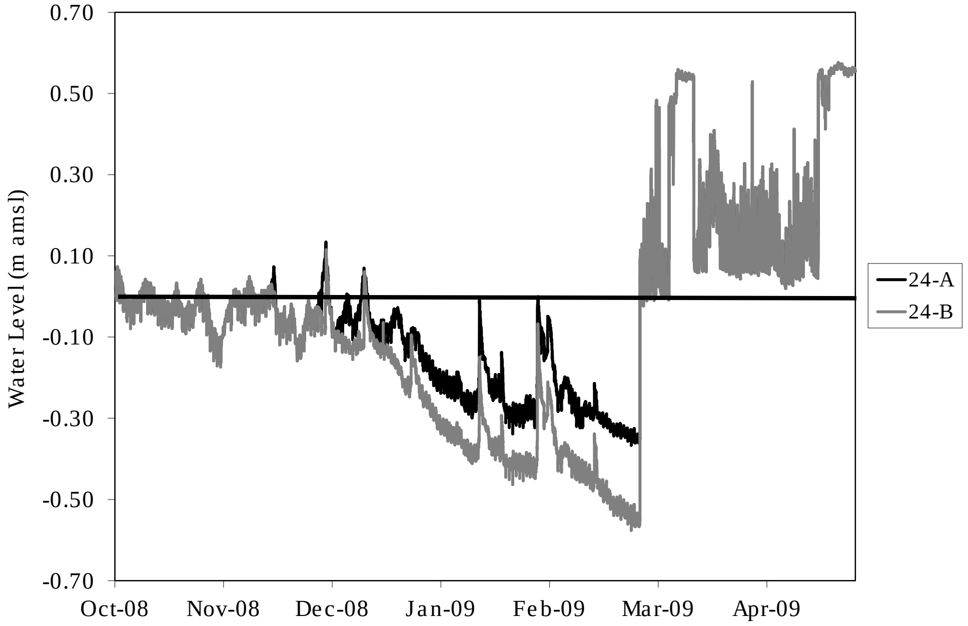

Supplementary Figure S9: Boxplots presenting dry bulk densities measured at the sampling sites in the dwarf (A), sparse (B) and dense (C) habitats in the non-managed and RIM impoundment in the periods 2008-2009 and 2013-2014. Different characters above the plots present significant differences ($p < 0.05$, Dunn's post hoc test) between the median values.

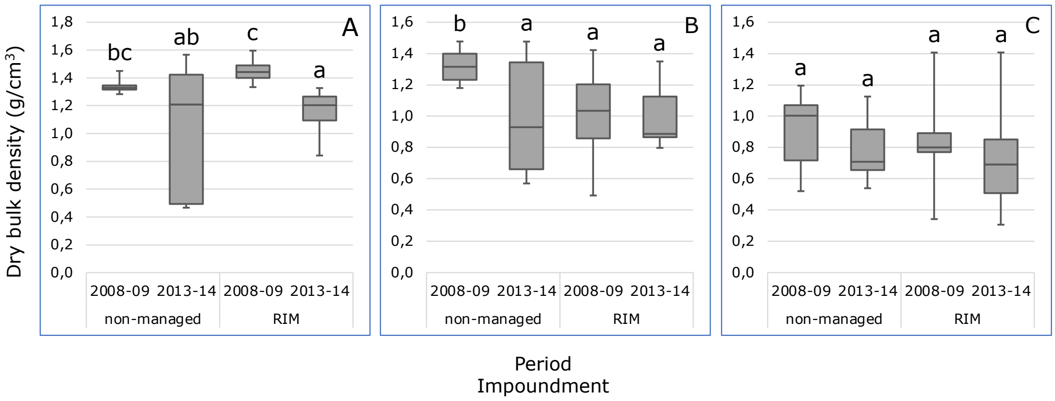

Note: No outliers were detected.

Supplementary Figure S10: Boxplots presenting concentrations of total nitrogen measured at the sampling sites in the dwarf (A). sparse (B) and dense (C) habitats in the non-managed and RIM impoundment in the periods 2008-2009 and 2013-2014. Different characters above the plots present significant differences (*p* < 0.05. Dunn's post hoc test) between the median values.

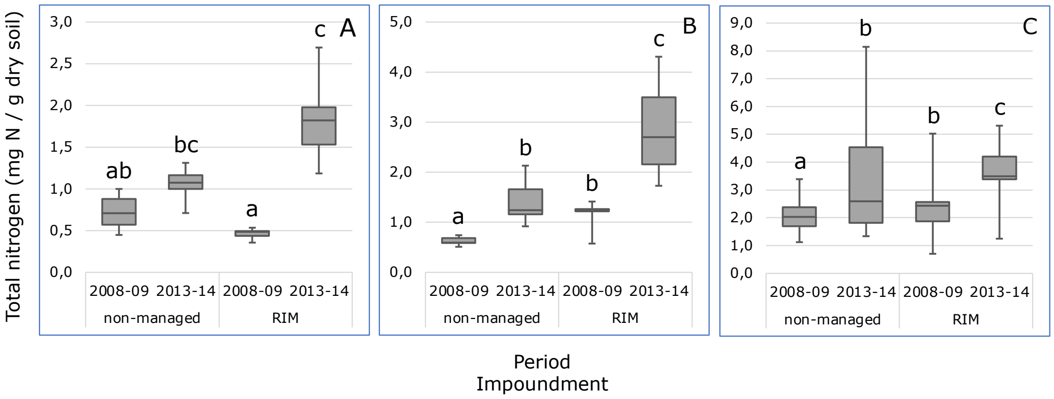

Note: One outlier was deleted in the dwarf habitat of the non-managed impoundment and one outlier in the dense habitat of the RIM impoundment, both period 2.

Supplementary Figure S11: Boxplots presenting concentrations of labile organic nitrogen measured at the sampling sites in the dwarf (A). sparse (B) and dense (C) habitats in the non-managed and RIM impoundment in the periods 2008-2009 and 2013-2014. Different characters above the plots present significant differences (*p* < 0.05. Dunn's post hoc test) between the median values.

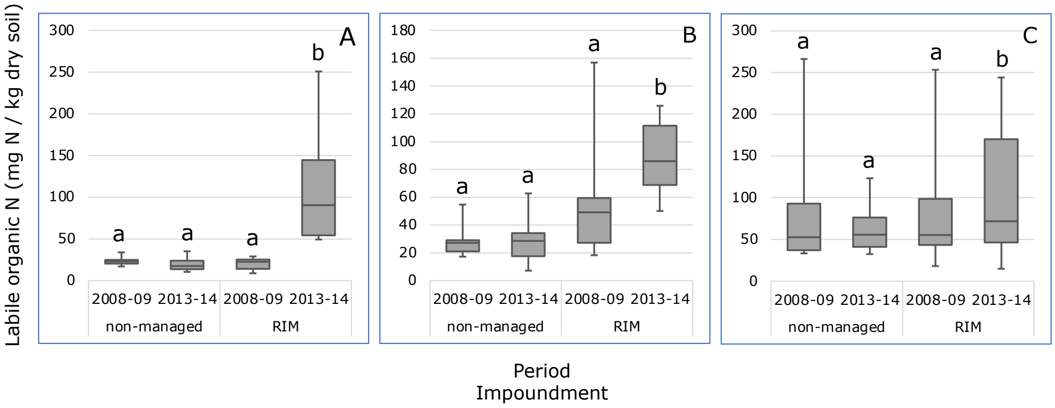

Note: No outliers were detected.

Supplementary Figure S12: Boxplots presenting salinity values measured at the sampling sites in the dwarf (A). sparse (B) and dense (C) habitats in the non-managed and RIM impoundment in the periods 2008-2009 and 2013-2014. Different characters above the plots present significant differences ($p < 0.05$. Dunn's post hoc test) between the median values.

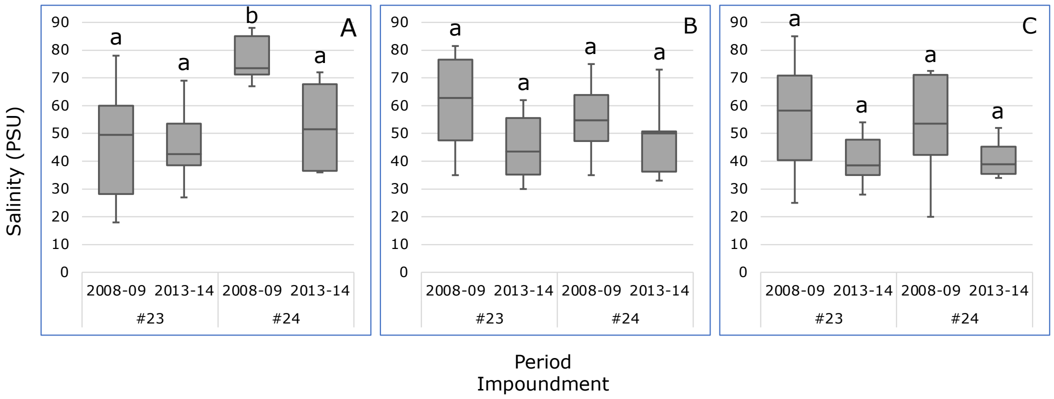

Note: No outliers were detected.
